# Supplementary material for: Recombinant Virus Quantification Using Single-Cell Droplet Digital PCR: A Method for Infectious Titer Quantification
Source: Viruses. 2023 Apr 26;15(5):1060. doi: 10.3390/v15051060 (PMC10221717; doi:10.3390/v15051060)
Supplement: Supplementary file 1 [file viruses-15-01060-s001.zip › viruses-2335707-supplementary.pdf]

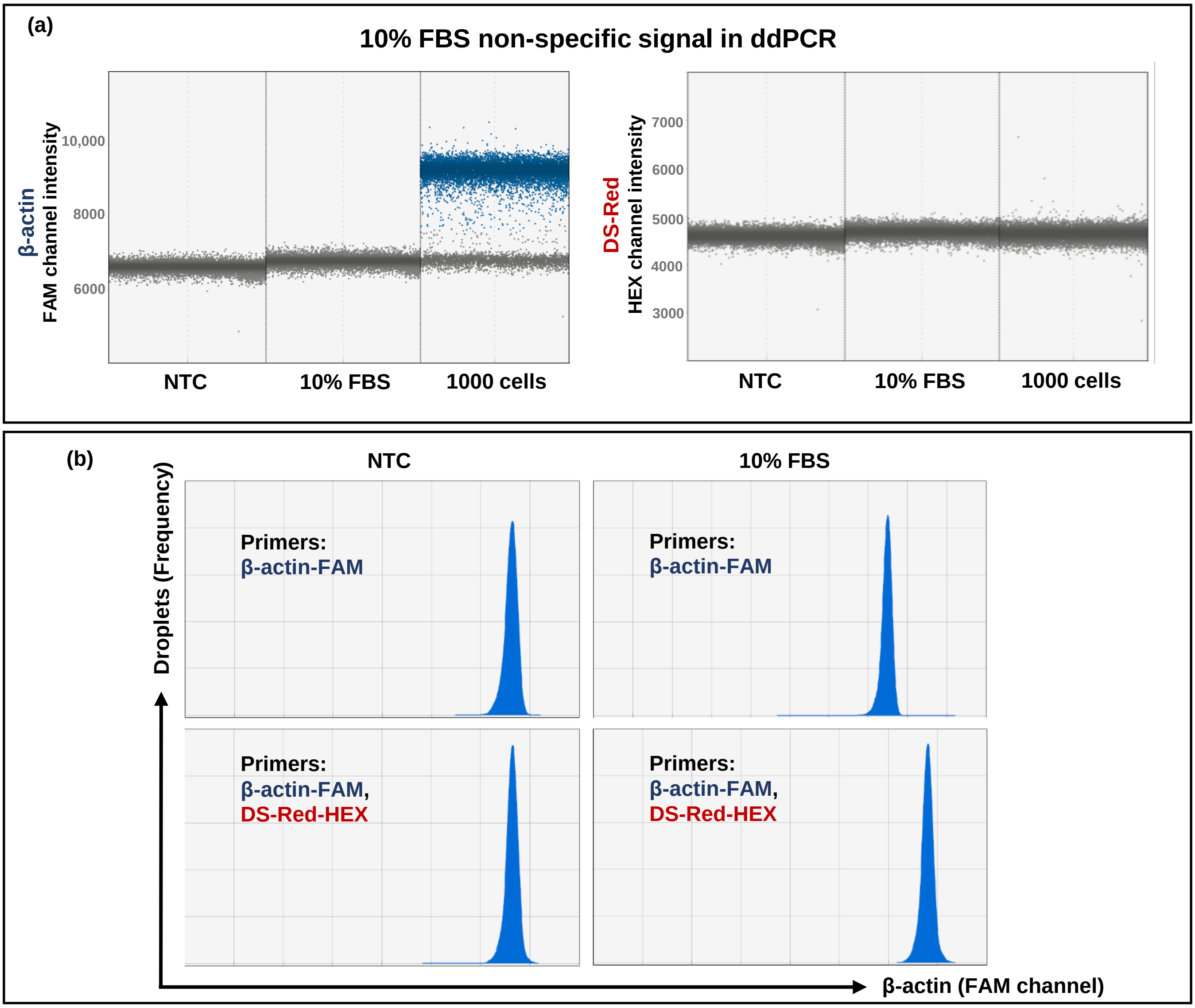

Figure S1. 10% FBS does not give a non-specific signal in ddPCR. **(a)** β-actin-FAM and DS-Red-HEX positive droplets of NTC, 10% FBS and 1000 BHK-21 cells; blue—droplets positive in FAM channel; grey—negative droplets. **(b)** Histogram of NTC and 10% PBS quantified using (i) only β-actin-FAM primers or (ii) β-actin-FAM primers in combination with DS-Red-HEX primers. NTC—non-template control.

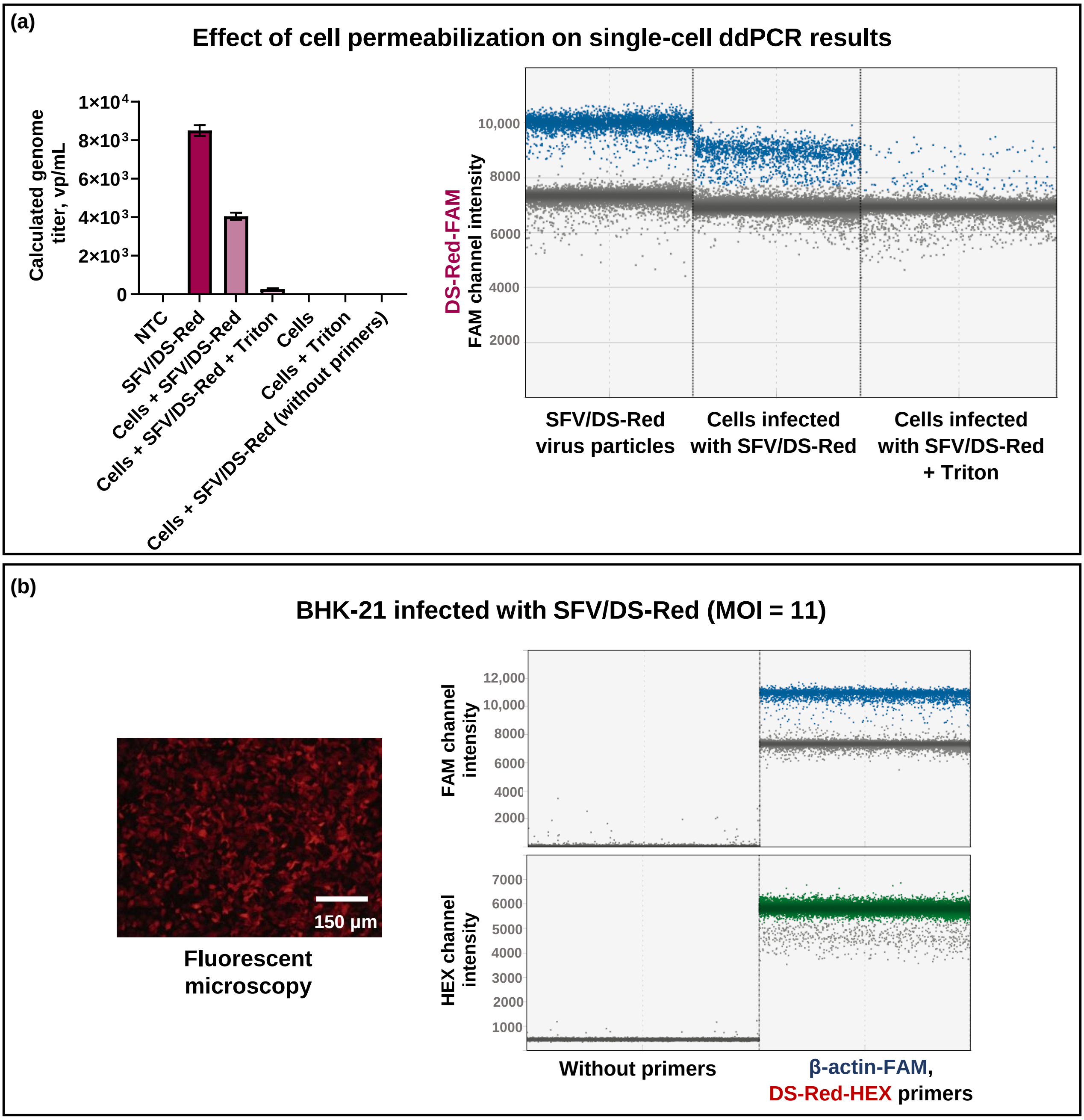

Figure S2. Optimization of infected cell ddPCR quantification. **(a)** Effect of cell permeabilization on single-cell ddPCR results. 4T1 cells were infected with SFV/DS-Red and used for ddPCR quantification with DS-Red_7816-FAM primers. 0.02% Triton was used for 4T1 cell permeabilization. **(b)** BHK-21 cells infected with SFV/DS-Red (MOI = 11) do not give an amplification signal without primers. In contrast, ddPCR quantification of the same sample with β-actin-FAM, DS-Red_7603-HEX primers provide a positive signal. NTC—non-template control; MOI—a multiplicity of infection; blue—droplets positive in a FAM channel; green—droplets positive in a HEX channel; grey—negative droplets. Results are presented as mean ± confidence interval.
